# Acute and mid-term outcomes of drug-coated balloon following rotational atherectomy

**DOI:** 10.1007/s12928-019-00611-y

**Published:** 2019-08-16

**Authors:** Taito Nagai, Masahiro Mizobuchi, Atsushi Funatsu, Tomoko Kobayashi, Shigeru Nakamura

**Affiliations:** Cardiovascular center, Kyoto Katsura hospital, 17 Yamada hirao-cho, Nishikyo-ku, Kyoto, Japan

**Keywords:** Stentless strategy, Drug-coated balloon, Rotational atherectomy

## Abstract

Rotational atherectomy (RA) is effective for reducing calcified plaque volume as part of percutaneous coronary intervention (PCI). Most lesions are then treated by stenting, but we often observe in-stent restenosis (ISR) due to an under-expanded stent associated with severe calcification, a condition that is particularly challenging to treat. It is unknown if drug-coated balloon (DCB) application following RA can be used as a “stentless” treatment strategy for calcified lesions. The aim of this study is to assess the acute and mid-term efficacy of DCB following RA (RA + DCB) at our institute and to evaluate the overall clinical utility of this stentless strategy for complex calcified lesions. From October 2014 to June 2018, 3644 lesions in 2424 consecutive cases were treated with PCI at our institute. Rotational atherectomy was used for 12.3% of all lesions and 42.3% of these RA-treated lesions were then treated using DCBs (*n* = 190 RA + DCB-treated lesions, of which 72% were in males). In-hospital major adverse cardiac events included only one case of non-Q-wave myocardial infarction. Average duration of follow-up coronary angiography after initial PCI was 199 ± 61 days. Angiographic restenosis was observed in 17.8% of RA + DCB-treated lesions, with mean late lumen loss of 0.23 ± 0.69 mm, while late lumen enlargement was observed in 39.1% of RA + DCB-treated lesions. At mid-term clinical follow-up, there were no cardiac deaths and target lesion revascularization rate was only 16.4%. Rotational atherectomy followed by DCB demonstrated acceptable acute and mid-term efficacy, suggesting that this stentless strategy may be an effective option for complex calcified lesions with high risk of ISR.

## Background

Percutaneous coronary intervention (PCI) for calcified lesions has not achieved satisfactory results compared to standard treatment, especially for small coronary vessel and diffuse lesions, even using drug-eluting stents (DES). Rotational atherectomy (RA) is a recently developed technique for calcified plaque debulking. In most cases, RA is followed by stenting. However, we often encounter in-stent restenosis (ISR) with under-expanded stents due to severe calcification. Drug-coated balloon (DCB) angioplasty has shown a very low target lesion revascularization rate of 2.3 − 6.7% in small vessels. In addition, DCB resulted in negative late lumen loss (late lumen enlargement) in 48% of lesions [1–3]. Despite these advantages, it is unknown if DCB following RA can be used as a stentless strategy for calcified lesions. The aim of this study is to examine acute and mid-term outcomes of DCB following RA at our institute as a potential stentless PCI strategy for complex calcified lesions.

## Methods

### Study population

**Fig. 1:**
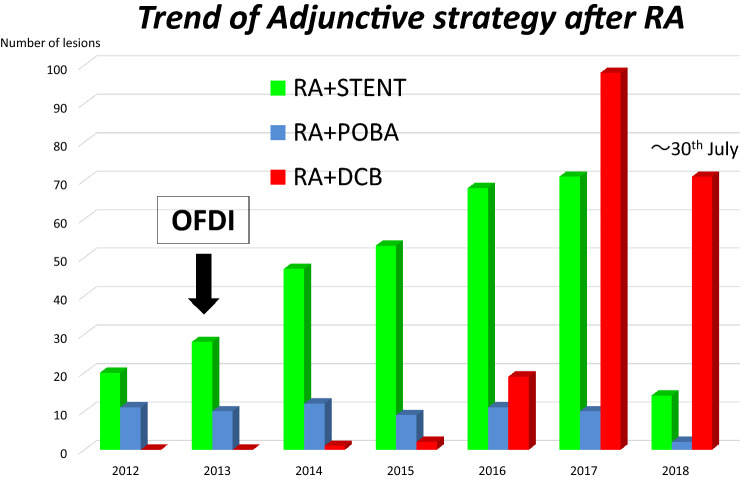
Trend of adjunctive strategy after rotational atherectomy at kyoto katsura hospital

**Fig. 2:**
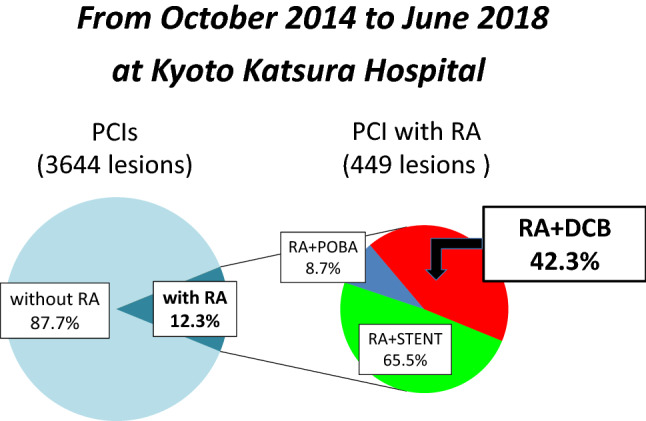
PCI strategy from october 2014 to june 2018 at kyoto katsura hoispital

Optical frequency domain imaging (OFDI) has greatly facilitated the use of RA for debulking calcified lesions. Thus, our current strategy is to use RA for ablation rather than mere plaque modification. We have also found better than expected results using DCB, and so have shifted adjunct therapy after RA from DES to DCB (Fig. 1). From October 2014 to June 2018, 3644 lesions in 2424 consecutive patients were treated by PCI at our institute. Rotational atherectomy was used for 12.3% of all lesions, and DCB was employed following 42.3% of these RA procedures (RA + DCB, *n* = 190). Specific details of the PCI strategy were at the discretion of the individual operators. Procedural, clinical, and angiographic data from these 190 RA + DCB-treated lesions were collected from medical records and retrospectively analyzed (Fig. 2). Coronary lesion type was categorized according to the American Heart Association/American College of Cardiology (AHA/ACC) classification.

### Procedure

All PCIs were performed with OFDI, optical coherence tomography (OCT), or intravascular ultrasound (IVUS) for intravascular imaging and for judging the debulking effect of RA. Rotational atherectomy was performed using the Rotablator (Boston Scientific, Natick, MA) at a rotational speed of 158000 ± 19000 rpm, maximum burr size of 1.8 ± 0.3 mm, and burr/artery ratio of 0.74 ± 0.17. Predilatation balloons after RA were selected by the operators (scoring balloon in 82% of cases and standard balloon in 14% of cases). Drug-coated balloons were used following RA and predilatation only in cases without flow-limiting dissection and severe vessel recoil. Ballooning after RA was performed relatively aggressive in cases that we originally planned to use DES, but we selected relatively low-pressure ballooning in cases that have been planned to use DCB. We intentionally selected a stentless strategy of using DCB following RA as possible to have optional treatment when restenosis occurs. In the study, 76.3% lesions were treated with DCB following RA even though it was possible to use DES, while 23.7% lesions were treated with DCB following RA because it was unsuitable for using DES. In this group, the main reasons for using DCB instead of stenting after RA were as follows: (1) target lesion was in a small vessel (53%), (2) ISR (29%), (3) insufficient debulking effect to get enough stent expansion because of severe calcification (20%), (4) diffuse disease (9%), and (5) others (7%).

Paclitaxel-coated balloons (SeQuent Please; B Braun, Melsungen, Germany) were used in the present study at the nominal pressure for 72 ± 25 s. The mean DCB diameter was 2.9 ± 0.4 mm. In the present study, no case required additional stenting after DCB (Table 1).

**Table 1:** Procedural data

Imaging device (%)	
OFDI or OCT	76
IVUS	24
RA wire (%)	
Floppy	45
Support	49
Both	6
Maximum burr size (mm)	1.8 ± 0.3
Burr/artery ratio	0.74 ± 0.17
Total number of burr	1.5 ± 0.5
Rotational speed (rpm)	158000 ± 19000
Dual antiplatelet therapy (%)	
Aspirin + ticlopidine	4
Aspirin + clopidogrel	22
Aspirin + prasugrel	74
Predilatation before DCB	
Type of balloon (%)	
Scoring balloon	82
Standard balloon	14
Balloon diameter (mm)	2.8 ± 0.5
Balloon length (mm)	12.5 ± 4.2
Dilation pressure (atm)	12 ± 4
DCB	
DCB diameter (mm)	2.9 ± 0.4
Total DCB length (mm)	26.2 ± 8.9
Dilation pressure (atm)	9 ± 4
Inflation time (s)	72 ± 25

### Quantitative coronary angiography (QCA)

Follow-up coronary angiography was scheduled at 6 months after PCI. Angiograms performed at baseline, at the end of PCI, and at follow-up were analyzed using QAngio XA version 7.3 (MEDIS Medical Imaging System BV, Leiden, The Netherlands). Angiographic restenosis was defined as ≥ 50% diameter block at the time of follow-up.

### Study endpoints and definitions

The study endpoints were occurrence of major adverse cardiac events (MACE), defined as the composite of death, Q-wave or non-Q-wave myocardial infarction (QMI or NQMI), and clinically driven target lesion revascularization (TLR). Myocardial infarction was defined as CK-MB elevation ≥ 10 times the upper limit of normal with (QMI) or without new abnormal Q-wave on the electrocardiogram (ECG) [4]. Angiographic results and restenosis rate were examined by qualitative comparative analysis (QCA). Late lumen enlargement was defined as negative late lumen loss.

## Results

### Patient and lesion characteristics

Patient characteristics are summarized in Table 2. A total of 190 lesions (in 167 patients) were treated with RA + DCB (mean age 75 ± 8 years, 72% male). A high proportion of these RA + DCB-treated patients had diabetes mellitus (56%) or chronic kidney disease requiring hemodialysis (21%). Left ventricular ejection fraction was 59% ± 13% and 7% of these cases had acute coronary syndrome. Lesion characteristics are summarized in Table 3. Of these lesions, 55% were in the left anterior descending (LAD) artery, 31% in the left circumflex (LCX) artery, and 14% in the right coronary artery (RCA). Most (74%) were type B2/C lesions, 73% were de novo, and 77% exhibited severe calcification.

**Table 2:** Patient characteristics

	*n* = 167
Age, years	75 ± 8
Male (%)	72
Hypertension (%)	84
Dyslipidemia (%)	59
Diabetes mellitus (%)	56
Family history (%)	14
History of smoking (%)	59
Hemodialysis (%)	21
Old myocardial infarction (%)	35
Prior bypass surgery (%)	8
Ejection fraction (%)	59 ± 13
Acute coronary syndrome (%)	7

**Table 3:**
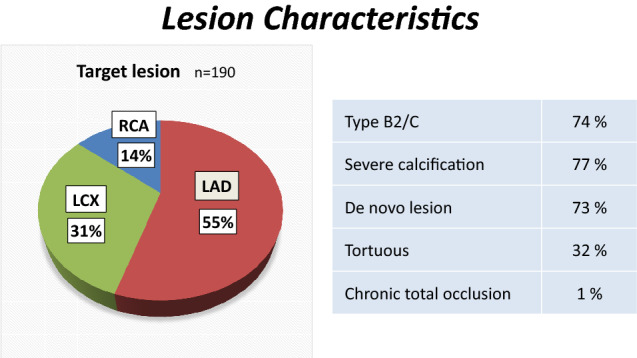
Lesion characteristics

### Angiographic data

**Fig. 3:**
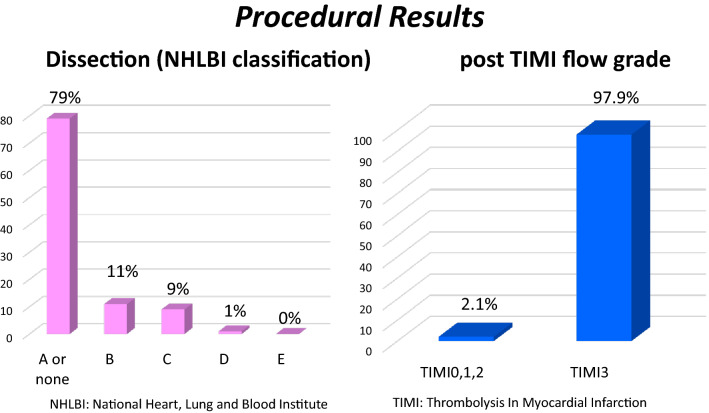
Angiographic results about dissection/TIMI flow grade

Angiographic results were categorized according to the National Heart, Lung and Blood Institute (NHLBI) definitions. Most were class A or non-dissecting, and 97.8% achieved thrombolysis in myocardial infarction (TIMI) flow grade 3 at the end of the procedure according to angiography (Fig. 3).

The QCA data are summarized in Table 4. Reference vessel diameter (RD) was 2.53 ± 0.60 mm before PCI and 2.67 ± 0.58 mm after PCI, while minimum luminal diameter (MLD) was 1.14 ± 0.49 mm before and 2.05 ± 0.59 mm after PCI, and % diameter stenosis (%DS) was 55.2% before and 23.3% after PCI. Lesion length was 17.0 ± 10.7 mm, and acute gain was 0.91 ± 0.50 mm.

**Table 4:** Quantitative coronary angiography (QCA)

	*n* = 190
Reference vessel diameter (mm)	
Pre	2.53 ± 0.60
Post	2.67 ± 0.58
Minimal luminal diameter (mm)	
Pre	1.14 ± 0.49
Post	2.05 ± 0.59
Diameter stenosis (%)	
Pre	55.2
Post	23.3
Lesion length (mm)	17.0 ± 10.7
Acute gain (mm)	0.91 ± 0.5
Mid-term follow-up	
Follow-up rate (eligible)	73% (87/119)
Average follow-up duration (days)	199 ± 61
Reference vessel diameter (mm)	2.74 ± 0.67
Minimal luminal diameter (mm)	1.90 ± 0.67
Diameter stenosis (%)	32.1
Late lumen loss (mm)	0.23 ± 0.69
Restenosis rate (%)	17.8
Late lumen enlargement (%)	39.1

Acceptable mid-term follow-up angiograms were obtained in 73% of cases (87/119 lesions, 107 patients). Median follow-up duration was 199 ± 61 days after the initial procedure. At follow-up, RD was 2.74 ± 0.67 mm, MLD was 1.90 ± 0.67 mm, and late lumen loss was 0.23 ± 0.69 mm. Angiographic restenosis was observed in 17.8% of lesions, and late lumen enlargement in 39.1% of lesions.

### Representative case

**Fig. 4:**
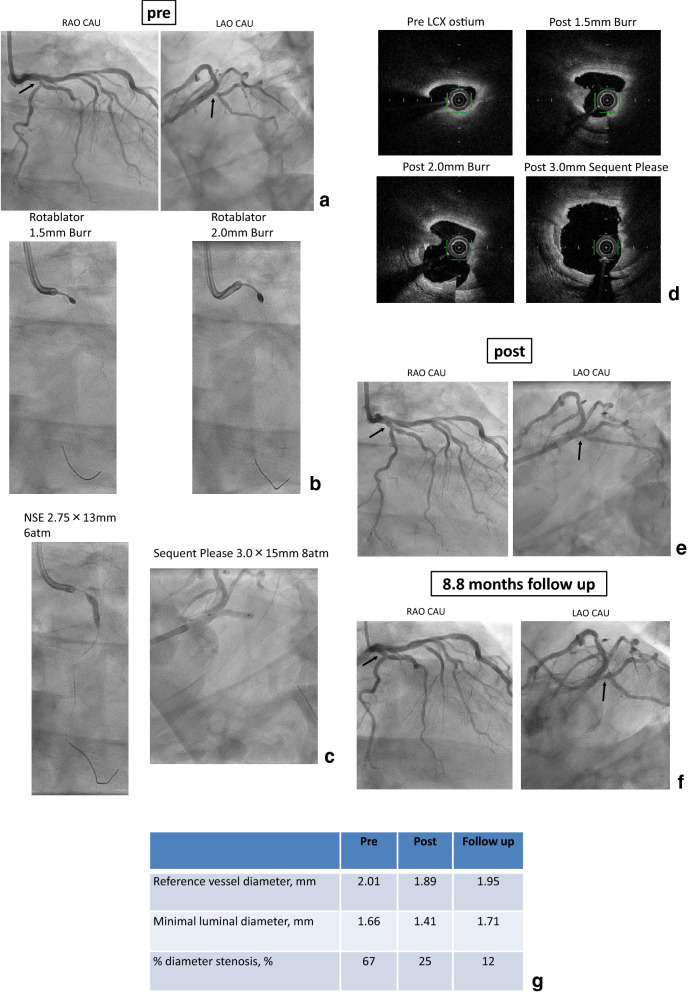
Representative case. **a** Pre angiography. **b** Rotablator, Burr size = 1.5, 2.0 mm. **c** NSE 2.75 × 13 mm (6 atm), Sequent Please 3.0 × 15 mm (8 atm). **d** OFDI findings (pre/post ablation 1.5 mm burr/post ablation 2.0 mm burr/post). **e** Post angiography. **f** 8.8 months follow up angiography. **g** QCA data (reference vessel diameter, minimal luminal diameter, % diameter stenosis)

A representative case is shown in Fig. 4. The patient was a 75-year-old male who had hypertension, diabetes mellitus, chronic kidney disease (HbA1c = 7.2%, eGFR = 50 ml/min/1.73 m^2^). He was admitted to our hospital with complaints of chest oppression and firstly LAD#7 was treated with DES following RA (Resolute integrity 2.75 × 18 mm, Medtronic, Santa Rosa, California). PCI was performed for LCX#11 ostium with severe calcification on another day. This severe calcified lesion was treated with RA (Burr size: 1.5 → 2.0 mm). The OFDI findings showed effective debulking results by RA and finally drug-coated balloon was added. Follow-up coronary angiography was performed at 8.8 months after the procedure and showed no restenosis. QCA date revealed late lumen enlargement and effective results of RA + DCB strategy (Fig. 4).

### Clinical data

In-hospital MACE included only one case of NQMI and no deaths, QMI, or TLR. During the mid-term clinical follow-up period (196 ± 37 days after initial PCI procedure), there were no cardiac deaths, and TLR rate was 16.4%. One patient was lost to clinical follow-up (Table 5).

**Table 5:** Clinical follow-up

In-hospital MACE	*n* = 167
Cardiovascular death (%)	0
Non-cardiovascular death (%)	0
QMI (%)	0
NQMI (%)	1
Target lesion revascularization (TLR) (%)	0
Mid-term clinical follow-up	
Average follow-up duration (days)	196 ± 37
Cardiovascular death, (*n*)	0% (0/107)
Non-cardiovascular death, (*n*)	2% (2/107)
Target lesion revascularization (TLR), (*n*)	16.4% (19/116)
Target vessel revascularization (TVR), (*n*)	20.7% (24/116)

### Predictors of TLR

The groups were compared using the Student’s *t* test and a multivariate logistic regression model was used to identify the predictors of TLR. The patient/lesion characteristics and the QCA findings were entered into the models. Stat View 5.0 (SAS Institute Inc, Cary, NC, USA) was used for statistical calculations. The results of multivariate analysis are shown in Table 6. In this analysis, there were no independent predictors of TLR.

**Table 6:** Predictors of TLR on multivariate analysis

Variables	Univariate OR [95% CI]	*p* value
Family history	0.059 [0.004 − 0.980]	0.0483
Post % diameter stenosis	1.185 [1.014 − 1.386]	0.0327

Variables	Multivariate OR [95% CI]	*p* value
Family history	0.044 [0.002 − 1.095]	0.0568
Post % diameter stenosis	1.186 [0.994 − 1.415]	0.0582

*OR* odds ratio, *CI* confidential interval

## Discussion

Mid-term outcomes following RA + DCB treatment for complex calcified lesions were acceptable, with no cardiovascular deaths, 16.4% TLR rate, and 17.8% restenosis rate. Previous studies have found TLR rates of 3.6 − 11.7% for DES following RA [5–10]. Although the TLR rate was higher in the present study using RA + DCB compared to RA + stenting, there are still substantial advantages to the stentless strategy. For instance, ISR tends to reoccur in small vessels, and a stentless strategy permits several treatment options in such cases, for example, additional debulking by RA. Similarly, for those cases in which we could not achieve sufficient debulking by RA (even using 2 burrs) and predicted stent under-expansion or residual balloon indentation at the lesion, a stentless strategy makes it possible to schedule a subsequent session using a larger burr. A previous study reported asymmetrical expansion in up to 50% of coronary stents deployed in calcified lesions [11]. There is general agreement that lesions of greater arc, length, or thickness are highly prone to stent under-expansion [12], and stent under-expansion is associated with an increase in ischemic events at 1 year [13]. DCB should be considered for lesions that are unsuitable for stenting, such as small-vessel and diffuse lesions or lesions with hinge motion. Before DCB was approved, we selected RA + POBA treatment for such lesions. Basically it is the meaning of the extension of POBA following RA as a procedure of DCB following RA; however, we have started to select stentless strategy with DCB because the performance of DCB following RA was better than expected. It is important to maintain as many options as possible for lesions at high risk of restenosis, and initial stenting limits additional PCI for recurrent ISR.

The DCBs used in this series inhibit intimal proliferation by delivering paclitaxel to the vessel wall, and have demonstrated effectiveness for ISR, small-vessel lesions, and diffuse lesions [1, 2, 14]. However, DCB has been reported to be ineffective for peripheral calcified lesions, likely because the drug does not reach the vessel wall due to the plaque barrier (although neointimal formation on the ablated calcified plaque may be reduced by DCB).

Drug-coated balloon treatment following RA should be used only in cases without flow-limiting and severe acute recoil after predilatation. Cortese et al. found that leaving non-flow-limiting dissections untreated after DCB is safe and not associated with the outcome. Indeed, Type A–C dissections were observed in their study, and almost all lesions treated with DCB were healed at follow-up angiography [15]. Funatsu et al. reported that 56% lesions of small vessel disease treated with DCB showed late lumen enlargement [16]. Even using the RA, there was no association between dissection type and outcome in our study. Additionally, RA can reduce calcified plaque volume and increase acute gain.

There are few reports on outcomes of DCB following RA for calcified lesions. Rissanen reported a very low TLR rate (1.5% at 12 months and 3.0% at 24 months) [17] compared to our study. One reason for this difference may be lesion selection. In our study, there were more small-vessel and diffuse lesions. In addition, more frequent routine follow-up angiography may lead to repeat interventions. About 70% of the reasons for performing TLR/TVR were that it was judged restenosis by follow-up coronary angiography (TLR: 13/19 lesions, TVR: 18/24 lesions). The other reason for performing TLR/TVR was the presence of clinical symptoms. Ito et al. reported a restenosis incidence of 13.9% at 6.5 months, late lumen loss of 0.03 mm, and late lumen enlargement of 47.2% after DCB for patients with de novo calcified coronary lesions using QCA (RA was used in 82% of cases), in accord with the present study. However, it should be noted that the present study enrolled restenosis cases including ISR as well as de novo lesion cases [18].

Intracoronary imaging modalities such as OFDI, OCT, and IVUS have facilitated PCI development by expanding our understanding of coronary calcium placing and wire bias. In particular, OFDI as a second-generation OCT modality with higher resolution than IVUS and OCT [19] may prove especially valuable for such applications. We intentionally use OFDI as possible; however, 24% of all cases were evaluated with IVUS in the study. We used IVUS in the case that the patients have a tendency of fluid retention like congestive heart failure or chronic kidney disease for preventing fluid load brought by OFDI use. Using intracoronary imaging modalities in all cases, every procedure was conducted safely and effectively.

### Study limitations

This is the first report investigating PCI outcomes using DCB following RA without stenting in our country. However, there are several limitations in the present observational study. First, this was a single-center, non-randomized, retrospective study with relatively small sample size and angiographic follow-up rate. Second, although dual antiplatelet therapy (DAPT) was performed in all cases, the duration of DAPT after PCI was at the discretion of each surgical team. In addition, the details of the procedure, such as the use of standard or scoring balloon, were also at the surgeon’s discretion. Furthermore, we did not enroll a comparative group such as stent placement following RA.

## Conclusions

Treatment of complex calcified lesion using DCB following RA yielded an acceptable TLR rate of 16.4%, with only one case of in-hospital MACE (NQMI). This stentless strategy is a potential new option for complex calcified lesion treatment. Indeed, we are currently shifting to RA use for calcified lesions as part of a debulking rather than plaque modification strategy.
